# The Impact of Normalization Procedures on Surface Electromyography (sEMG) Data Integrity: A Study of Bicep and Tricep Muscle Signal Analysis

**DOI:** 10.3390/s25092668

**Published:** 2025-04-23

**Authors:** Sergio Fuentes del Toro, Josue Aranda-Ruiz

**Affiliations:** 1 Department of Mechanical Engineering, University Carlos III of Madrid, Avda. de la Universidad 30, 28911 Leganés, Spain; 2 Department of Continuum Mechanics and Structural Analysis, University Carlos III of Madrid, Avda. de la Universidad 30, 28911 Leganés, Spain; jaranda@ing.uc3m.es

**Keywords:** surface electromyography, normalization methods, muscle activation, sports, rehabilitation

## Abstract

Surface electromyography (sEMG) is a critical tool for quantifying muscle activity and inferring biomechanical function, enabling the detection of neuromuscular deficits through the analysis of electrical potential propagation. However, the inherent variability in sEMG signal amplitude, influenced by factors such as electrode placement, equipment characteristics, and individual physiology, necessitates robust normalization techniques for accurate comparative analysis. This study investigates the reliability and effectiveness of several normalization methods in the context of bicep and tricep muscle activation during dynamic and isometric exercises: maximum voluntary contraction (MVC), submaximal voluntary contraction (SMVC), remote voluntary contraction (RVC), mean, and peak normalization. We conducted a comprehensive experimental protocol involving healthy volunteers, capturing sEMG signals during controlled bicep curls, tricep extensions, and isometric contractions. The efficacy of each normalization method was evaluated based on its ability to minimize inter-subject variability and enhance signal consistency. Specifically, while SMVC, MVC, and RVC methods exhibited generally superior performance in normalizing bicep and tricep signals, the optimal method varied depending on the task and muscle, providing consistent and reliable data for biomechanical analysis. These results underscore the importance of selecting appropriate normalization techniques to improve the accuracy of sEMG-based assessments in clinical and sports biomechanics, contributing to the development of more effective rehabilitation protocols and performance enhancement strategies.

## 1. Introduction

Surface electromyography (sEMG) is a widely utilized technique in biomechanics for assessing muscle activation patterns during movement and other tasks. It has applications spanning sports performance, rehabilitation, and clinical assessments, providing non-invasive insights into neuromuscular function [1]. However, interpreting sEMG signals can be challenging due to inherent variability caused by individual differences, such as muscle size, activation levels, electrode placement, and skin impedance [2]. These factors, along with intra-individual variability influenced by fatigue, motor learning, and daily fluctuations in muscle activation [3], can obscure the detection of subtle yet clinically relevant changes. Therefore, normalization techniques are crucial to mitigate these variations and enable accurate comparisons across tasks, subjects, and conditions.

Normalization in sEMG aims to reduce inter-individual and inter-experimental variability by referencing the signal to a predefined standard. Among the most widely employed methods is maximal voluntary contraction (MVC) normalization [4], which scales the signal to the peak amplitude observed during an isometric maximal contraction. This approach has been extensively validated and is considered a gold standard for assessing muscle activation levels [5,6,7].

However, achieving true maximal activation can be challenging. Factors such as fatigue, posture, and individual motivation can influence maximal effort [8,9,10,11,12], making MVC normalization less feasible for certain populations, such as older adults or individuals with neurological conditions, due to variability in motor performance [13,14]. The underlying rationale for using MVC rests on the assumption that in healthy individuals, a maximal voluntary effort results in near-maximal or truly maximal neural drive to the muscle, leading to a force output that approximates the muscle’s physiological limit [15]. Consequently, the EMG activity recorded during this maximal effort is often considered a reliable indicator of the muscle’s full activation potential [16]. However, this assumption is not universally valid and can be particularly compromised in populations experiencing muscle fatigue or those with neuromuscular disorders [15]. Furthermore, training background and muscle conditioning also play a crucial role in influencing muscle activation patterns across different contraction modalities [17]. For instance, strength-trained individuals may exhibit more linear EMG-moment relationships and better control of muscle activation compared to untrained individuals [18]. Different types of warm-up exercises, such as isotonic or isometric contractions, can also lead to variations in muscle activity and performance during subsequent exercises [19]. Furthermore, the stability of the surface during contractions can affect force output and EMG activity, with unstable conditions potentially leading to decreased force but similar or even increased EMG activity in stabilizing muscles [20]. The neural drive and motor unit recruitment strategies also play a critical role, with factors such as contraction type affecting discharge rates and the ability to achieve full voluntary activation [21,22]. As summarized in Table 1, MVC normalization requires maximum effort, which may not be feasible in all situations.

Submaximal voluntary contraction (SMVC) normalization addresses some of the limitations of MVC by using a percentage of the maximum effort as the reference point, typically 70% [12,23,24]. This method is particularly advantageous in scenarios where maximal effort may compromise data quality or participant safety, such as in clinical or elderly populations or in athletic settings where repeated maximal effort may not be feasible [12,23,24]. Similarly, remote voluntary contraction (RVC) normalization is designed for populations where maximal contractions are not feasible, such as older adults or individuals with clinical conditions, and allows for the detection of changes in neural conductivity [23]. However, as also shown in Table 1, submaximal contractions may not account for inter-individual variability in muscle activation levels, potentially leading to less reliable normalization [14].

In contrast to static methods like MVC and SMVC, dynamic normalization methods, including mean and peak-based normalization, offer practical solutions for tasks requiring continuous motion, such as gait or running analyses. These methods are less demanding and avoid the fatigue associated with sustained isometric contractions, making them suitable for sports performance analysis [25,26,27]. However, dynamic methods are sensitive to movement inconsistencies and baseline noise, necessitating careful selection of time windows and preprocessing techniques [28,29]. Despite these challenges, studies have shown that dynamic normalization can provide more reliable results than static methods in certain applications [30,31]. Table 1 summarizes the descriptions and challenges of these common sEMG normalization techniques.

**Table 1:** Comparison of common sEMG normalization techniques, their descriptions, and associated challenges based on existing literature.

Normalization Technique	Description	Challenges
MVC	Involves recording the EMG signal during a maximum voluntary isometric contraction and using this value as a reference.	Requires maximum effort and may not be feasible for individuals with motor impairments or pain [13,32].
Peak	Normalizes the EMG signal to the peak electrical activity observed during a specific task.	Task-specific and may not be suitable for comparing muscle activation across different tasks [33].
SMVC	Normalizes the EMG signal to a submaximal reference value, such as 60% of the maximum voluntary contraction.	May not account for inter-individual variability in muscle activation levels [32].
Dynamic	Uses a reference value derived from the task itself, such as the first repetition of a set or the peak activation during a movement.	Provides more reliable results in task-specific applications [30,31].

Recent studies comparing normalization methods have shown that their effectiveness varies depending on the task and population. For example, 50% MVC normalization was effective for gait analysis in healthy individuals [34], while a ratio of MVC to RVC was more appropriate for spinal cord injury patients during cycling [35]. In the context of rehabilitation, RVC normalization proved beneficial for Parkinson’s patients during fine motor tasks [36], whereas MVC normalization was optimal for children with cerebral palsy performing isometric tasks [37].

Given the variety of normalization methods available and their differing applications, it is crucial to understand the advantages and limitations of each method to ensure accurate interpretation of sEMG data. Unlike previous studies, which often focused on isolated normalization methods, this work aims to evaluate multiple methods under controlled experimental conditions. The goal is to determine how different normalization techniques influence the interpretation and reliability of sEMG signals, particularly in tasks relevant to sports and rehabilitation. This study focuses on the biceps and triceps muscles as these are commonly studied in the context of upper-limb movement and are critical in both athletic and rehabilitative exercises.

The interpretation of surface electromyography (sEMG) signals is inherently complex due to significant inter-individual variability arising from factors such as muscle size, subcutaneous fat, and electrode placement [2], as mentioned earlier. Furthermore, intra-individual variability, influenced by fatigue, motor learning, and daily fluctuations in muscle activation, can obscure the detection of subtle yet clinically relevant changes over time [3]. A critical challenge in sEMG analysis lies in selecting appropriate normalization techniques to mitigate these variabilities and enable meaningful comparisons across individuals and experimental conditions. Many conventional normalization methods exhibit task-specificity, limiting the generalizability of findings across different movements and populations. This is particularly pertinent in clinical and rehabilitative settings, where the ability of patients to perform maximal voluntary isometric contractions (MVICs), a common normalization reference, may be compromised [16,32]. To address these limitations, this study aims to systematically evaluate the reliability and effectiveness of a range of normalization methods, including both traditional and dynamic approaches [31], when applied to the biceps and triceps muscles during dynamic and isometric tasks. By comparing their performance, we seek to provide evidence-based guidance for researchers and practitioners in selecting normalization strategies that are sensitive to subtle changes in muscle activation, robust across different individuals and tasks, and practically applicable in diverse settings, including clinical and rehabilitative contexts.

Our analysis revealed that the most appropriate normalization method depends on several factors, including the type of muscle activity and the population under study. MVC is the most widely used method, but SMVC and normalization to external forces or moments can also be effective in certain situations [38]. Some limitations and challenges associated with each normalization method were identified, such as the difficulty of obtaining reliable MVC measurements in some populations and potential errors in normalizing to external forces or moments [28,29].

This study seeks to contribute to this body of knowledge by systematically evaluating multiple normalization methods applied to the bicep and tricep muscles. By comparing these techniques under controlled conditions, we aim to provide a framework for selecting normalization methods tailored to specific research and clinical applications. Specifically, the primary objective of this study is to systematically evaluate the reliability and effectiveness of various sEMG normalization methods, focusing on their application to dynamic tasks involving the bicep and tricep muscles. This work aims to address critical gaps identified in the current literature by comparing the performance of multiple normalization methods—including MVC, SMVC, RVC, and dynamic approaches—across different exercises and muscle groups; investigating the applicability of these methods to dynamic, sport-specific, and rehabilitative contexts; exploring inter-individual variability and its impact on signal reliability; and contributing to the standardization of normalization protocols.

Through this analysis, the study seeks to provide actionable insights for researchers and practitioners, guiding the selection of normalization methods tailored to specific research questions, muscle groups, and practical applications in sports and rehabilitation. By bridging these gaps, this work aims to enhance the reliability, interpretability, and clinical utility of sEMG data.

## 2. Materials and Methods

Following the research questions outlined in the introduction, an experiment was designed to analyze surface electromyography (sEMG) signals from the bicep and tricep muscles in both arms. Additionally, the digitorum and carpi radialis muscles from both sides were monitored to facilitate remote voluntary contraction (RVC) measurements. In total, eight muscles were assessed in each volunteer. The study was approved by the ethics committee of the Universidad Carlos III de Madrid, ensuring adherence to ethical research standards.

Prior to the experimental tasks, each volunteer was thoroughly informed about the study’s objectives and potential risks. They received a detailed document outlining the procedures and signed an informed consent form, with the assurance that participation was voluntary and they could withdraw at any time. Subsequently, participants completed a short questionnaire collecting anthropometric data, including age, height, and weight, to document inter-individual variability.

For this study, a convenience sample of healthy, physically active young adults was recruited. The participant group comprised 25 volunteers: 14 males (mean age: 27 ± 5 years, mean body mass: 75 ± 10 kg, and mean height: 178 ± 7 cm) and 11 females (mean age: 25 ± 3 years, mean body mass: 62 ± 8 kg, and mean height: 165 ± 6 cm). All participants reported engaging in regular sports or physical activity at least twice a week, indicating a generally good level of physical fitness.

Sensor placement followed a standardized protocol to ensure accurate data acquisition. A palpation test was conducted to locate the muscle belly, and the area was prepared by shaving and cleaning with alcohol. Wireless sEMG sensors (Delsys Trigno+ system, USA) were placed on the muscles, adhering to the SENIAM guidelines for electrode placement. Special care was taken to minimize impedance and cross-talk from neighboring muscles.

Volunteers performed a series of four distinct exercises, each targeting different aspects of muscle activity:

- Exercise 1 (MVC): Maximal voluntary contraction (MVC) was assessed using isometric contractions of the biceps and triceps. Participants maintained their elbow at a 90-degree angle with their upper arms stationary and aligned naturally. Each contraction was held for 5 s and repeated three times, with 2 min of rest between repetitions. This exercise required 100% of the muscle’s maximal force, and the protocol ensured that compensatory movements, such as bending the back or moving the shoulders, were avoided.
  The MVC exercises for the biceps and triceps were conducted under controlled conditions to ensure accurate and consistent data collection. For the biceps, participants maintained their arms at a 90-degree elbow flexion, with a neutral wrist position. They performed an isometric contraction by flexing their biceps against a stationary dynamometer, exerting maximal force for 5 s.
  For the triceps, participants extended their elbows from a similar starting position, pushing against a rigid resistance to achieve maximal force output. Proper stabilization of the shoulder joint was ensured throughout both exercises to isolate muscle activity and prevent compensatory movements. Each MVC trial was repeated three times, with a two-minute rest period between repetitions to minimize fatigue and enhance reliability. The peak sEMG signal from each trial was recorded and used as a reference for normalization.
- Exercise 2 (SMVC): Submaximal voluntary contraction (SMVC) was performed similarly to Exercise 1 but at 70% of the maximal force, measured using a dynamometer. Volunteers followed the same positioning and contraction protocol, with 5-s contractions repeated three times and 2 min of rest between repetitions. This exercise was designed to assess muscle activity under moderate force levels, which are more representative of submaximal effort in real-life tasks.
- Exercise 3 (Isokinetic): Isokinetic contractions were recorded using an isokinetic dynamometer to control the contraction speed and range of motion. Sequential contractions of the right biceps, left biceps, right triceps, and left triceps were performed. The movement was constrained to a predefined range of motion and performed at a constant angular velocity. This exercise aimed to capture muscle activation patterns during dynamic, controlled movements under standardized conditions.
- Exercise 4 (Dynamic and RVC): A combination of dynamic and remote voluntary contraction (RVC) assessments was employed. Dynamic contractions involved controlled bicep curls and tricep extensions using a 4 kg weight, with the range of motion standardized between 30 and 120 degrees of elbow flexion. Simultaneously, isometric contractions of the digitorum and carpi radialis were recorded. Each dynamic contraction was performed slowly and consistently to minimize variability, while the isometric contractions were held for 5 s. This combined approach allows for the evaluation of muscle activity in dynamic tasks while capturing neuromuscular control of remote muscle groups.

Each exercise was repeated three times, with a 5 min rest interval between exercises to prevent fatigue and ensure reliable data collection. The protocol was designed to minimize the risk of injury while capturing consistent and high-quality sEMG signals. This comprehensive experimental setup allows for a robust comparison of different normalization methods across various muscle groups and contraction types, providing insights into their applicability in sports and rehabilitation contexts.

### 2.1. Instrumentation

**Figure 1:**
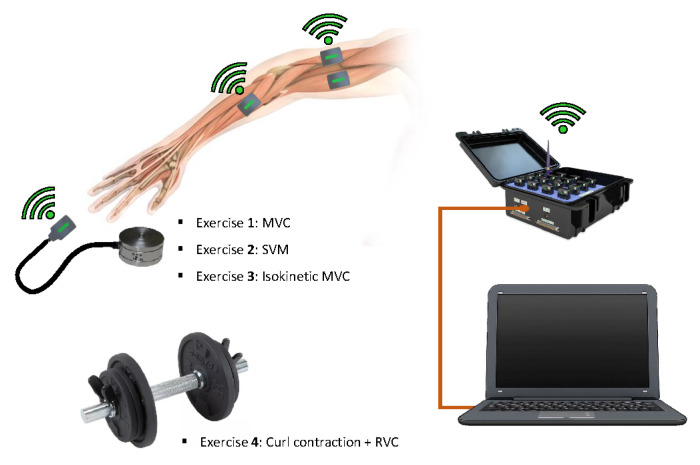
Instrumentation used in the experiment and exercises performed by each volunteer. The wireless EMG system connected to a recording computer for data acquisition. Wireless EMG sensors are shown placed on arm muscles (biceps and triceps), along with a wireless analog output system for measuring force. A dumbbell used for dynamic exercises is also depicted. The four exercise modalities performed in the study are listed as follows: maximal voluntary contraction (MVC), submaximal voluntary contraction (SVM), isokinetic contraction, and remote voluntary contraction (RVC).

The instrumentation used in this study can be divided into two categories: devices for measuring muscle activity and devices for measuring forces. These tools were carefully selected to ensure accurate data collection and reproducibility of results, as depicted in Figure 1.

For measuring muscle activity, a superficial electromyography (sEMG) system was utilized. This technology, previously validated in similar studies [39,40], allows for precise wireless measurement of muscle signals to prevent movement artifacts because of cable and muscle interference [41].

The robustness of our sEMG measurements was a key consideration in this study. Several potential sources of noise can affect sEMG signals, including inherent electrode noise, movement artifacts, electromagnetic interference, cross-talk from neighboring muscles, and internal physiological signal instability. To mitigate these factors, we employed a Delsys Trigno+ wireless sEMG system with integrated sensors utilizing silver contacts, which are known for their stable electrical properties and adequate signal-to-noise ratio [42]. Electrodes were carefully placed on the muscle belly after preparing the skin by shaving and cleaning with alcohol to minimize skin impedance and potential cross-talk [43,44]. Movement artifacts, often caused by cable movement or relative motion between the skin and electrodes [45], were minimized by the integrated wireless design of the Trigno+ system. Electromagnetic noise, primarily power-line interference, was addressed through offline processing using a band-pass filter with cutoff frequencies of 40 Hz and 400 Hz, a range recommended in previous sEMG studies [40,46,47,48]. Cross-talk was further reduced by the sensor dimensions (27 × 37 × 13 mm) and careful bipolar spacing, as suggested by Winter et al. [49]. Finally, while inherent signal instability due to motor unit firing rates (0–20 Hz) is a characteristic of sEMG [50], our analysis focused on frequency components within the 40–400 Hz range, minimizing the direct impact of this lower-frequency variability on the normalized amplitude measures. By implementing these measures and preprocessing steps, we aimed to ensure the acquisition of robust and reliable sEMG signals suitable for normalization and subsequent analysis.

In total, eight muscles were monitored for each volunteer: the biceps and triceps of both arms, as well as the digitorum and carpi radialis, which were essential for the remote voluntary contraction (RVC) protocol. The sensor placement adhered to SENIAM [5] guidelines to ensure consistency and reproducibility across participants, aligning with best practices in sports and rehabilitation research.

For measuring forces, a calibrated hand dynamometer was used to determine both the maximal voluntary contraction (MVC) force and the 70% contraction force (SMVC) required for submaximal efforts. The dynamometer provided real-time feedback, enabling participants to maintain the required force levels during isometric and isokinetic exercises. In addition, free weights were employed for the dynamic exercises. Participants performed controlled bicep and tricep curls with a standardized weight of 4 kg. This combination of tools ensured comprehensive data collection across all exercise protocols, capturing both neuromuscular activity and force generation dynamics.

The selected instrumentation reflects the dual focus of this study: the sEMG system provided high-resolution insights into muscle activation patterns, while the force-measuring tools allowed precise quantification of exertion levels. Together, these systems ensured the reliability of data across different normalization methods, enhancing the applicability of the findings in sports performance analysis and rehabilitation interventions.

### 2.2. Data Analysis

The data collected from the sEMG sensors underwent a multi-step analysis process to evaluate the effectiveness of different normalization methods. This process included filtering the raw signals, applying various normalization techniques (MVC, SMVC, RVC, and dynamic methods), and performing statistical comparisons across exercises and volunteers. The analysis aimed to assess the reliability and consistency of each normalization method while accounting for inter-individual variability and the specific characteristics of each muscle group.

To address the inherent noise in sEMG signals, a preprocessing step was implemented. These signals are prone to environmental interference, cross-talk from neighboring muscles, and electromagnetic noise. A band-pass filter with a lower cutoff frequency of 40 Hz and an upper cutoff frequency of 400 Hz was applied, as suggested by previous studies [40,46,47,48]. This filtering step ensured that the data were clean and suitable for subsequent normalization and analysis.

**Figure 2:**
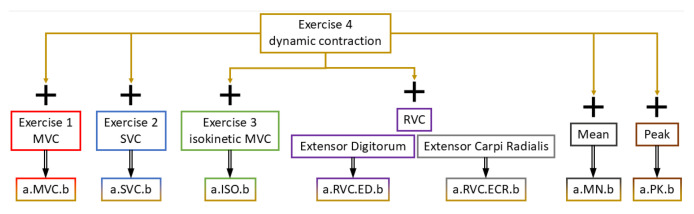
Normalization process of the sEMG signal (a: exercise (CT: curl triceps and CB: curl biceps) and b: side of the exercise (R: right and L: left).

Following the filtering process, the signals were normalized using seven different techniques: MVC, SMVC, SMVC, and RVC (from the carpi radialis and digitorum muscles), as well as the peak and mean of the filtered signal. These normalization methods provided a quantitative assessment of muscle activation during the exercises. Figure 2 illustrates the normalization workflow, using an example from the right tricep during a bicep curl exercise. The nomenclature in the figure specifies the exercise performed (CB: curl biceps or CT: curl triceps), the normalization method used (e.g., MVC, SMVC, ISO for isokinetic MVC, PK for peak, MN for mean, and RVC for remote voluntary contraction), and the side of the body analyzed (R: right, L: left).

**Figure 3:**
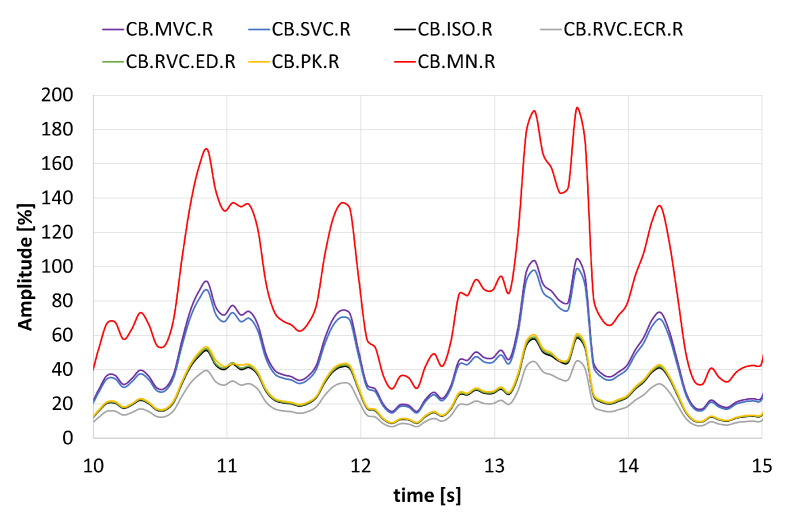
Example of a five-second segment of the normalized signals from the right biceps of a single volunteer during a bicep curl exercise.

To demonstrate the impact of different normalization methods, Figure 3 shows an example of normalized signals from the right biceps of a single volunteer during a bicep curl exercise. The graph highlights the differences in signal amplitudes, particularly during the contraction phase, which occurs around the 11th and 12th seconds.

The analysis involved comparing the normalized signals with the original data using several statistical measures. These included the following:

- Cross-Correlation Coefficient (CCC): A measure of the similarity between two signals, defined in Equation (1):
  (1) $$\mathrm{CCC}=\frac{n\left(\sum xy\right)-\left(\sum x\right)\left(\sum y\right)}{\sqrt{\left[n\left(\sum x^{2}\right)-{\left(\sum x\right)}^{2}\right]\left[n\left(\sum y^{2}\right)-{\left(\sum y\right)}^{2}\right]}}.$$
- Linear Correlation Coefficient (LCC): Evaluates the strength and direction of the linear relationship between two variables, as shown in Equation (2):
  (2) $$\mathrm{LCC}=\frac{\sum \left(x-\bar{x}\right)\left(y-\bar{y}\right)}{\sqrt{\sum {\left(x-\bar{x}\right)}^{2}\sum {\left(y-\bar{y}\right)}^{2}}}.$$
- Spearman Correlation Coefficient (SPCC): A non-parametric measure for assessing monotonic relationships between two variables, defined in Equation (3):
  (3) $$\mathrm{SPCC}=1-\frac{6\sum D^{2}}{N\left(N^{2}-1\right)}.$$
  Here, *D* is the difference in the ranks of corresponding variables, and *N* is the number of data points.
- R-squared (R^2^): Indicates how well the regression model explains the variability in the observed data, as defined in Equation (4):
  (4) $$R^{2}=\frac{\sigma_{xy}^{2}}{\sigma_{x}^{2}\sigma_{y}^{2}}.$$

These statistical measures were used to evaluate the reliability of each normalization method and its ability to preserve the original signal characteristics. By systematically comparing the outcomes, this study provides a comprehensive assessment of the normalization techniques, offering insights into their suitability for different muscles and exercise types.

Moreover, to confirm that different normalization methods preserved the signal’s shape, we employed a two-step process. First, each signal was normalized to unit amplitude, ensuring all signals had a comparable amplitude scale. This was achieved by dividing each point of the signal by its maximum absolute value, as defined in Equation (5):

(5) $$x_{normalized}\left(t\right)=\frac{x(t)}{\max(|x(t)|)}.$$

Subsequently, the energy of each normalized signal was calculated as the integral of the squared signal over the time interval from $t_{initial}$ to $t_{final}$, as defined in Equation (6):

(6) $$Energy=\int_{t_{initial}}^{t_{final}}x_{normalized}{\left(t\right)}^{2}dt.$$

If all normalization methods resulted in the same energy value, it would imply that the signal’s shape was consistently preserved across all methods, regardless of variations in amplitude or duration.

## 3. Results

Prior to conducting a detailed statistical analysis, an assessment of signal shape similarity across all normalization methods and volunteers was performed. By normalizing each signal to unit amplitude and subsequently calculating its energy as the integral of the squared signal, we confirmed that all normalization methods resulted in identical energy values for each signal. This finding indicates that regardless of the specific normalization technique applied, the underlying shape of the sEMG signals remained consistent. This preliminary analysis ensured that subsequent statistical comparisons would focus on the effectiveness of the normalization methods in terms of correlation and variance explanation, rather than differences in signal shape.

To further evaluate the impact of the normalization procedures on data quality, we assessed the Signal-to-Noise Ratio (SNR) for the original sEMG signal and each of the normalization methods. The mean SNR for the raw sEMG signal was 2.77 ± 0.81 dB. Following normalization, the SNR values for the different methods remained largely consistent with the original signal. Specifically, CARA presented an SNR of 2.99 ± 1.08 dB (108% of the original), DIGI 2.65 ± 0.80 dB (96%), ISOK 3.39 ± 0.76 dB (122%), MEAN 2.68 ± 0.74 dB (97%), PEAK 2.91 ± 0.94 dB (105%), and SMVC 3.12 ± 0.64 dB (113%). These results indicate that the normalization processes applied in this study did not introduce significant noise or substantially alter the signal quality relative to the original sEMG recordings.

This study aimed to evaluate the reliability and effectiveness of various sEMG normalization methods (MVC, SMVC, ISOK, CARA, DIGI, MEAN, and PEAK) across different exercises, muscle groups (biceps and triceps), and body sides (right and left). The analysis focused on comparing original and normalized sEMG signals using statistical metrics: the Concordance Correlation Coefficient (CCC), Spearman’s Correlation Coefficient (SPCC), Coefficient of Determination ($R^{2}$), and Linear Correlation Coefficient (LCC). Table 2 summarizes the mean and standard deviation of these metrics, providing a comprehensive overview of each normalization method’s performance.

The Concordance Correlation Coefficient (CCC) revealed moderate agreement between normalized and original signals. For the biceps, CCC values ranged from −0.028 (SMVC) to 0.689 (DIGI) on the right side and from 0.091 (PEAK) to 0.822 (CARA) on the left, with CARA showing the highest concordance. In the triceps, CCC values were generally lower, ranging from −0.12 (SMVC) to 0.52 (CARA) on the right, and from −0.08 (MEAN) to 0.602 (CARA) on the left, again with CARA performing best. These results suggest that CARA consistently provided the highest concordance, particularly for the left biceps and triceps, while DIGI was most effective for the right biceps.

Spearman’s Correlation Coefficient (SPCC) indicated strong linear relationships between normalized and original signals. For the biceps, SPCC values were high across all methods, with perfect correlations (ρ=1) for SMVC, ISOK, and MEAN on the right side, and a near-perfect correlation (0.994) for MEAN on the left. In the triceps, several methods, including SMVC, ISOK, and MEAN, achieved perfect correlations (ρ=1). Overall, this indicates that most normalization methods have a strong linear relationship with peak MVC values, especially for the triceps.

The Coefficient of Determination ($R^{2}$) showed that the normalization methods effectively explained the variability in EMG data. For the biceps, DIGI showed the highest $R^{2}$ value (0.793) on the right side, and MEAN had an extremely high $R^{2}$ value (0.999) on the left. In the triceps, several methods, including SMVC, ISOK, and MEAN, achieved perfect $R^{2}$ values (1), indicating strong explanatory power. These findings suggest that MEAN and MVC methods are particularly effective in explaining the variance in EMG signals.

The Linear Correlation Coefficient (LCC) showed significant variability, especially in the triceps. For the biceps, SMVC and ISOK showed relatively high LCC values on the right, and MEAN exhibited a significantly high LCC value (742.2) on the left. In the triceps, high LCC values were observed for several methods but with greater variability. This variability, particularly in the biceps, suggests sensitivity to individual muscle characteristics.

**Figure 4:**
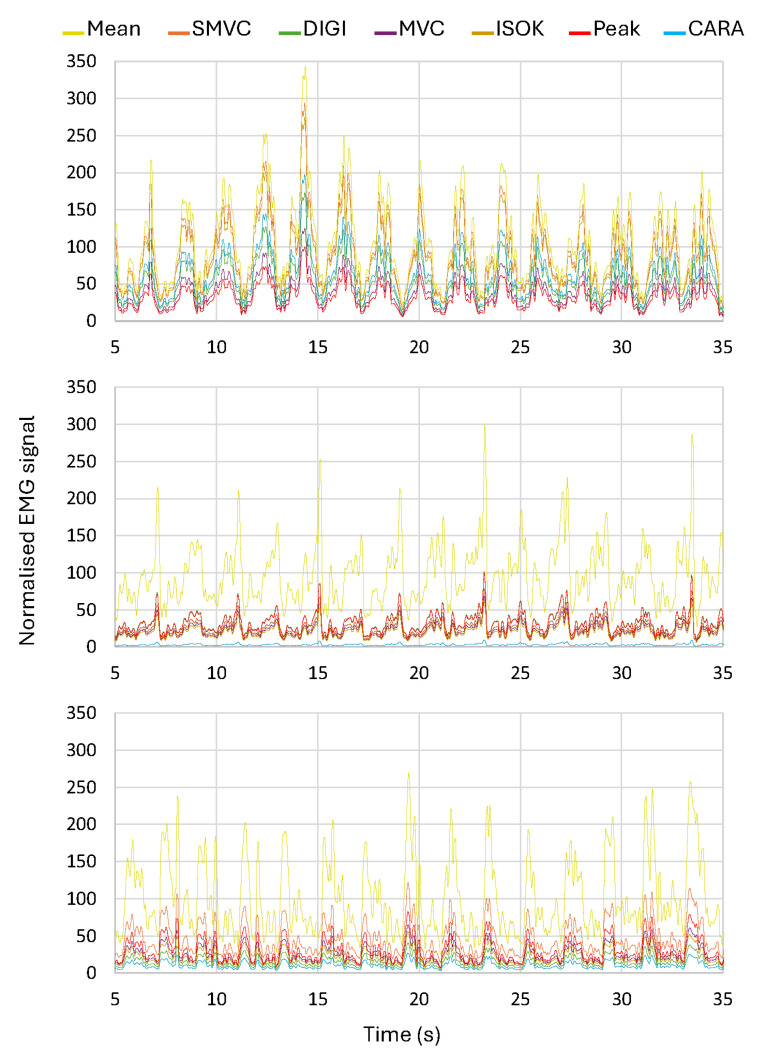
Comparison of normalized sEMG signal amplitudes for the left tricep muscle of three different volunteers over a 30 s time window. The signals for each volunteer are displayed for seven different normalization techniques (yellow: MEAN, orange: SMVC, green: DIGI, wine: MVC, gold: ISOK, red: PEAK, and blue: CARA), highlighting the inter-individual variability and temporal dynamics of the normalized muscle activity.

To illustrate the impact of different normalization methods and to provide insight into the inter-individual variability and temporal dynamics of the normalized sEMG signals, Figure 4 presents data from the left tricep muscle of three different volunteers over a 30 s window. This expanded visualization allows for a comparison of the normalized signal amplitudes across the seven different normalization methods (MEAN, SMVC, DIGI, MVC, ISOK, PEAK, and CARA) for each of the three individuals over the specified time course. The inclusion of data from multiple subjects and a longer duration provides a more comprehensive representation of the variability observed in the normalized sEMG signals during dynamic muscle activity.

**Figure 5:**
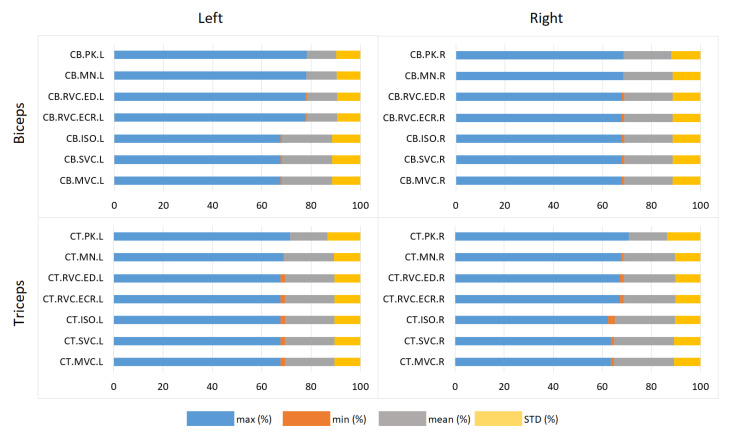
Distribution of statistical metrics across normalization methods for bicep and tricep muscles during curl exercises.

Additionally, Figure 5 displays the distribution of statistical metrics (maximum, minimum, mean, and standard deviation) for each normalization method, providing a comprehensive view of their performance across exercises and body sides. This visualization aids in understanding the consistency and variability of each method.

In summary, no single normalization method consistently outperformed others across all conditions. CARA normalization was most reliable for the left biceps and triceps, while DIGI excelled in the right biceps. MEAN normalization was effective in dynamic tasks, particularly in terms of $R^{2}$. These results underscore the need for tailored normalization strategies based on specific muscle groups and activity contexts, emphasizing the importance of careful method selection in biomechanical studies.

Table 3 summarizes the sensitivity (mean Concordance Correlation Coefficient, CCC) and effectiveness (maximal Coefficient of Determination, $R^{2}$) of each normalization method across muscles, compared to prior studies. Sensitivity was computed as the mean CCC between normalized and original signals across all muscles (in Equation (7)) [51,52]:

(7) $$\mathrm{Sensitivity}=\frac{\sum {\mathrm{CCC}}_{\mathrm{right}\;\mathrm{biceps}}+{\mathrm{CCC}}_{\mathrm{left}\;\mathrm{biceps}}+{\mathrm{CCC}}_{\mathrm{right}\;\mathrm{triceps}}+{\mathrm{CCC}}_{\mathrm{left}\;\mathrm{triceps}}}{4},$$

while effectiveness reflected the maximal $R^{2}$ value per method [16,53].

CARA normalization exhibited superior sensitivity (0.63±0.04) and effectiveness ($R^{2}=0.82$) for the left biceps and triceps, outperforming MVC in dynamic tasks, consistent with task-specific normalization principles. SMVC showed lower sensitivity (−0.02±0.23) than MVC, contradicting earlier isometric-focused studies, likely due to dynamic task interference. ISOK demonstrated moderate effectiveness ($R^{2}=0.52$) for controlled movements but limited utility in multi-joint contexts. MVC explained substantial variance ($R^{2}=0.85$) for the triceps but overestimated activation in dynamic tasks, aligning with critiques of its task specificity. MEAN normalization achieved near-perfect variance explanation ($R^{2}=1.0$), supporting its use for dynamic signal trends. DIGITORUM normalization is a novel approach; as such, there is no direct precedent. However, our findings highlighted stabilizer interference, as in [54].

These results emphasize that CARA and MEAN normalization provide advantages for dynamic and multi-muscle studies, whereas MVC remains suitable for isolated isometric tasks. Method selection must consider movement complexity and stabilizer engagement to ensure valid sEMG interpretations.

## 4. Discussion and Conclusions

This study aimed to evaluate the effectiveness of various normalization methods for surface electromyography (sEMG) signals in analyzing muscle activity during dynamic and isometric tasks. By comparing seven normalization techniques across multiple metrics (CCC, SPCC, $R^{2}$, and LCC), we provide insights into their reliability and application, particularly for the bicep and tricep muscles (Table 4). The results are crucial for understanding muscle function in contexts such as sports performance and rehabilitation.

### 4.1. Discussion of Key Findings

The findings underscore the variability in the effectiveness of normalization methods depending on the muscle, side of the body, and type of activity:

1. CARA and DIGI Normalizations: The CARA (remote voluntary contraction from carpi radialis) method demonstrated the most consistent results across metrics for the left biceps and the left triceps, achieving the highest CCC and SPCC values. This aligns with prior studies suggesting that CARA normalization is particularly effective for tasks involving coordinated upper-limb activities [55]. Similarly, DIGI normalization was most effective for the right biceps, indicating its potential utility in asymmetric or dynamic tasks. These methods may be advantageous in rehabilitation scenarios where maximal effort is difficult to achieve, such as for individuals with neuromuscular impairments.
2. MEAN and MVC Normalizations: The MEAN normalization method excelled in dynamic tasks, with near-perfect $R^{2}$ scores for the left biceps. This result highlights its ability to preserve the overall variance and energy of the signal, making it a reliable choice for activities involving fluctuating muscle activation. On the other hand, MVC normalization consistently yielded high correlations across all metrics, reinforcing its position as a gold standard in sEMG analysis, especially in controlled, isometric conditions [5]. However, the reliability and validity of MVC as a normalization reference can be influenced by various factors, even in healthy individuals. The force produced during an MVC can exhibit variability within the same individual across different trials due to motivation levels, perceived pain, learning effects, and variations in testing protocols [12]. Achieving a consistent and true MVC can be challenging, affecting the reliability of normalized EMG data and the detection of genuine changes in muscle activation [12]. Furthermore, the characteristics of the MVC task, such as the contraction type and joint angle, can significantly impact the recorded MVC value, potentially leading to inaccurate normalization when applied to different activities [9,10]. A notable limitation is the potential for exceeding 100.
3. Variability Across Muscles and Sides: Significant differences were observed between muscles and body sides. The triceps exhibited lower CCC and SPCC values compared to the biceps, particularly for the right side. This could reflect inherent differences in muscle architecture or activation patterns, as well as greater sensitivity to electrode placement and inter-individual variability. These findings emphasize the need to tailor normalization methods to the specific muscle group under analysis.
4. Implications for Sports and Rehabilitation: In sports performance analysis, where dynamic and repetitive movements are common, MEAN and CARA methods emerge as practical choices due to their robustness in preserving signal characteristics under varying conditions. In rehabilitation, particularly for clinical populations unable to perform maximal contractions, DIGI and CARA methods offer viable alternatives, allowing for meaningful comparisons without necessitating exhaustive effort.

### 4.2. Limitations and Future Directions

While this study provides valuable insights, several limitations should be acknowledged:

- Sample Size: The study was conducted on a limited number of volunteers. Expanding the sample size and including individuals from diverse populations (e.g., athletes, elderly individuals, and patients with musculoskeletal disorders) would enhance the generalizability of the findings.
- Exercise Variability: The analysis focused on specific tasks involving the biceps and triceps. Incorporating a broader range of exercises, including those targeting lower-limb muscles, would provide a more comprehensive assessment of normalization methods.
- Dynamic Noise and Artifacts: Despite filtering, residual noise, or cross-talk may have influenced the results. Future work could explore advanced signal processing techniques, such as adaptive filtering or machine learning-based noise reduction.
- Real-World Applications: Although the methods were tested under controlled conditions, further studies are needed to validate their effectiveness in real-world scenarios, such as during sports training or clinical assessments.
- Limitations of MVC: As discussed in the previous section, the reliability of MVC can be compromised by factors such as participant motivation, pain, learning effects, and the specific testing conditions [9,10,12,23]. This is particularly relevant in cases of muscle fatigue [8] and neuromuscular disorders [5], where the ability to generate a true maximal contraction is often impaired. Future research should further investigate alternative normalization methods that are less reliant on maximal effort, especially for clinical populations.
- Antagonistic Muscle Co-activation: While the study design aimed to isolate agonist muscle activation during specific exercises, the potential influence of low-level antagonistic co-activation (biceps and triceps) on the recorded sEMG signals and normalization effectiveness was not explicitly analyzed. Future research should investigate the co-activation patterns and their impact on sEMG normalization across different contraction conditions by simultaneously recording and analyzing both agonist and antagonist muscle activity.
- Performance of Normalization Methods and Physiological Considerations: The effectiveness of different sEMG normalization methods was evaluated by comparing their statistical properties against the original, unnormalized signals across various muscles and contraction types. Table 2 provides a comprehensive summary of these comparisons, highlighting the strengths and weaknesses of each method. The Concordance Correlation Coefficient (CCC), which assesses both the correlation and the agreement in means between the normalized and original signals, revealed that CARA (remote voluntary contraction from carpi radialis) generally provided the highest agreement, particularly for the left biceps and triceps. This suggests that normalizing to a remote, submaximal isometric contraction of a forearm muscle might better preserve the overall shape and amplitude characteristics of the sEMG signal during both isometric and dynamic tasks of the upper arm, potentially because it is less susceptible to the variability and fatigue associated with maximal voluntary efforts of the target muscles themselves [16,56,57].
  The Spearman’s Correlation Coefficient (SPCC) consistently indicated strong linear relationships for most normalization methods, suggesting that the scaling applied by these techniques generally preserved the monotonic relationship within the signals. Notably, SMVC (submaximal voluntary contraction) and ISOK (isokinetic) often showed perfect correlations, implying a highly consistent scaling relative to the original signal’s trends [54,58]. Similarly, MEAN normalization, which scales the signal by its own average, also demonstrated near-perfect correlations, particularly for the left biceps and triceps, indicating a strong preservation of the signal’s temporal dynamics relative to its own central tendency [31].
  The Coefficient of Determination ($R^{2}$), representing the proportion of variance in the original signal explained by the normalized signal, was highest for MEAN normalization, especially for the left biceps and triceps. This indicates that scaling by the mean of the dynamic signal was most effective in capturing the overall variability inherent in the muscle activity [54]. MVC (maximal voluntary contraction) also showed good variance explanation, particularly for the triceps, suggesting that scaling to the maximal isometric capacity can effectively account for a substantial portion of the signal’s variability, especially in that muscle group [16]. The Linear Correlation Coefficient (LCC) exhibited the highest variability across methods and muscle groups, suggesting that the linear scaling factor between normalized and original signals was not consistent. This variability highlights the potential for different normalization methods to alter the absolute amplitude relationships within and between signals. Considering the potential physiological rationales, the consistent performance of CARA might be attributed to its provision of a stable, submaximal reference that is less influenced by the task-specific recruitment patterns and fatigue associated with maximal efforts of the biceps and triceps. The strong linear relationships observed with SMVC and ISOK suggest that scaling by a consistent submaximal or controlled dynamic reference can effectively preserve the overall signal trends. The high variance explained by MEAN normalization is likely due to its inherent adaptation to the specific characteristics of the dynamic signal itself. Conversely, the variability in LCC underscores that different normalization strategies can lead to different interpretations of the absolute levels of muscle activation [57,59]. Ultimately, the choice of normalization method should be carefully considered based on the specific research question and the characteristics of the muscles and tasks being investigated.
- Reliability and reproducibility of neuromuscular measurements: The reliability and reproducibility of neuromuscular measurements are critical considerations when evaluating muscle performance. While our study focuses on the effects of different normalization methods on EMG data, it is important to acknowledge that the reliability and reproducibility of the underlying muscle performance measurements themselves (upon which normalization is based) also play a significant role. Table 1 provides a summary of some of the challenges associated with these measurements. Maximal voluntary contraction (MVC) normalization, a widely used technique, relies on a measurement that is inherently susceptible to variability. As noted in Table 1 and supported by previous research [13,32], MVC requires maximal effort, which can be influenced by a range of factors, including subject motivation, pain, fatigue, and learning effects. These factors can compromise the reproducibility of MVC measurements, potentially leading to inconsistencies in normalized EMG data. Submaximal voluntary contraction (SMVC) normalization, while avoiding the need for maximal exertion, introduces its own challenges to reproducibility. The consistency of submaximal effort can be difficult to control, both within and between individuals, leading to potential variability in the normalized EMG signals. Factors such as changes in attention or perceived exertion can affect the reproducibility of SMVC-based normalization. Isokinetic testing offers a controlled dynamic measurement, aiming to improve reliability compared to less constrained movements. However, as highlighted in Table 1, isokinetic measurements are task-specific. The results obtained during a particular isokinetic test (e.g., at a specific angular velocity) might not be fully reproducible or generalizable to other dynamic tasks with different movement patterns or speeds. The influence of factors such as muscle fatigue and the specificity of the testing device can also affect reproducibility. In contrast, remote voluntary contraction (RVC), specifically the CARPI method used in our study, seeks to provide a more stable reference by normalizing to a contraction of a muscle that is not the primary mover in the task of interest. This approach aims to minimize the impact of fatigue and task-specific factors that can affect the reproducibility of MVC or dynamic measurements. However, the reproducibility of RVC measurements can still be influenced by factors such as electrode placement and individual variations in muscle activation patterns. In conclusion, each muscle performance measurement method (MVC, SMVC, Isokinetic, and RVC) has inherent limitations that can affect its reliability and reproducibility. A comprehensive evaluation of these factors requires careful consideration of the specific research context and the implementation of standardized measurement protocols.

**Table 4:** Statistical performance and potential physiological rationale for different normalization methods.

Normalization Method	Concordance (CCC)	Linear Relationship (SPCC)	Variance Explained ($R^{2}$)	Linear Correlation (LCC)	Overall Performance Summary	Potential Physiological Rationale for Performance
MVC	Moderate agreement, high variability in triceps.	Strong linear relationship across muscles.	Good variance explanation, especially for triceps.	High variability, particularly in triceps.	Generally good linear relationship and variance explanation, but agreement (CCC) can be moderate, especially in the triceps. High LCC variability.	May perform well when dynamic recruitment closely scales with maximal isometric capacity. Variability might arise if dynamic tasks involve different synergistic contributions or fatigue patterns not reflected in the MVC.
SMVC	Low to moderate agreement, best for right biceps.	Perfect correlation in biceps (right) and triceps.	Perfect variance explanation in triceps, good in biceps.	High LCC in right biceps, variable in triceps.	Strong linear relationship and variance explanation, but agreement (CCC) can be low for some muscles.	Using a submaximal isometric reference might be more reliable if maximal effort is inconsistent or if dynamic tasks are performed at submaximal levels. However, it might not capture the full range of motor unit recruitment during high-intensity dynamic actions.
ISOK	Low to moderate agreement, better for right biceps.	Perfect correlation in biceps (right) and triceps.	Perfect variance explanation in triceps, good in biceps.	High LCC in right biceps, variable in triceps.	Similar to SMVC in terms of strong linear relationship and variance explanation, but agreement (CCC) can be low for some muscles.	Normalizing to an isokinetic contraction (dynamic but at constant velocity) might better reflect recruitment during controlled dynamic movements. However, the specific velocity and contraction type of the isokinetic reference might not generalize to other dynamic tasks (like RVC).
CARA	Best overall agreement (CCC), especially left.	High linear relationship, consistent in both muscles.	Good variance explanation, robust across muscles.	Moderate variability, relatively consistent.	Most consistent method for signal agreement (CCC), good linear relationship and variance explanation across both biceps and triceps.	A remote, low-intensity isometric contraction might provide a more stable reference that is less influenced by the maximal effort variability and fatigue associated with MVC, potentially leading to better agreement with the shape of dynamic signals, even if the overall activation levels differ.
DIGI	Moderate agreement, best for right biceps.	High linear relationship, particularly for triceps.	Good variance explanation for biceps.	Moderate variability.	Good agreement for the right biceps, strong linear relationship, and good variance explanation for the biceps.	Similar rationale to CARA. The choice of a different remote muscle might capture some individual differences in neural drive or overall activation strategies that correlate better with specific dynamic tasks in certain individuals or limbs.
MEAN	Low to moderate agreement.	Near-perfect to perfect correlation, especially left biceps and triceps.	Highest variance explanation for left biceps and triceps.	High LCC for left biceps, variable elsewhere.	Strongest linear relationship and variance explanation, particularly for the left side. Agreement (CCC) can be lower compared to CARA.	Normalizing to the mean of the dynamic signal itself inherently captures the characteristics of the dynamic recruitment pattern. The high correlation and variance explained suggest it scales well with the overall activation changes, but it does not reference an external physiological maximum.
PEAK	Low agreement, especially left side.	High linear relationship, good for triceps.	Good variance explanation for triceps.	Moderate variability.	Generally good linear relationship and variance explanation for the triceps, but agreement (CCC) is often low, especially on the left side.	Normalizing to the peak of the dynamic signal focuses on the maximal activation achieved during that specific movement. This might be relevant if the research question centers on peak activation levels, but it does not provide a consistent external reference across different tasks or individuals.

### 4.3. Conclusions

This study aimed to evaluate the effectiveness of various normalization methods for surface electromyography (sEMG) signals in analyzing muscle activity during dynamic and isometric tasks. By comparing seven normalization techniques across multiple metrics (CCC, SPCC, $R^{2}$, and LCC), we provide insights into their reliability and application, particularly for the bicep and tricep muscles. The results are crucial for understanding muscle function in contexts such as sports performance and rehabilitation.

The findings underscored the variability in the effectiveness of normalization methods depending on the muscle, side of the body, and type of activity. The CARA (remote voluntary contraction from carpi radialis) method demonstrated the most consistent results across metrics for the left biceps and left triceps, achieving the highest CCC and SPCC values. This aligns with prior studies suggesting that CARA normalization is particularly effective for tasks involving coordinated upper-limb activities [55]. Similarly, DIGI normalization was most effective for the right biceps, indicating its potential utility in asymmetric or dynamic tasks. These methods may be advantageous in rehabilitation scenarios where maximal effort is difficult to achieve, such as for individuals with neuromuscular impairments. The MEAN normalization method excelled in dynamic tasks, with near-perfect $R^{2}$ scores for the left biceps. This result highlights its ability to preserve the overall variance and energy of the signal, making it a reliable choice for activities involving fluctuating muscle activation. On the other hand, MVC normalization consistently yielded high correlations across all metrics, reinforcing its position as a gold standard in sEMG analysis, especially in controlled, isometric conditions [5]. However, its dependency on maximal effort poses challenges in clinical or fatigued populations. Significant differences were observed between muscles and body sides. The triceps exhibited lower CCC and SPCC values compared to the biceps, particularly for the right side. This could reflect inherent differences in muscle architecture or activation patterns, as well as greater sensitivity to electrode placement and inter-individual variability. These findings emphasize the need to tailor normalization methods to the specific muscle group under analysis. In sports performance analysis, where dynamic and repetitive movements are common, MEAN and CARA methods emerge as practical choices due to their robustness in preserving signal characteristics under varying conditions. In rehabilitation, particularly for clinical populations unable to perform maximal contractions, DIGI and CARA methods offer viable alternatives, allowing for meaningful comparisons without necessitating exhaustive effort.

The selection of an appropriate normalization method in muscle activation analysis involves a critical consideration of the trade-off between reliability and variability. Dynamic normalization methods, while offering the advantage of being task-specific, present a complex profile in terms of these two factors. On the one hand, they demonstrate strong reliability in preserving the overall trend of muscle activation. This is evident in the consistently high Spearman Correlation Coefficients (SPCCs) observed between the RMS signal and dynamic normalization methods. For instance, the SPCC between the RMS and Peak reached 1.000 in both the left bicep and left tricep muscles, indicating an excellent preservation of the monotonic relationship, which is crucial for accurately reflecting the timing and general pattern of muscle activity changes. This suggests that dynamic methods robustly capture the temporal dynamics of muscle activation, a key aspect of reliability. 

However, dynamic methods introduce a notable degree of variability, particularly in the scaling of the normalized signal’s amplitude. This variability is reflected in the wider range of Linear Correlation Coefficients (LCCs) compared to static methods. For example, the LCC between the RMS and Mean varied substantially across muscles, ranging from −0.028 in the right bicep to 0.371 in the left bicep, demonstrating that the linear relationship between the normalized and original signals is not consistent across different muscles or, potentially, across different trials or subjects. This inconsistency in scaling can complicate the quantitative comparison of muscle activation levels. Furthermore, the R-squared (R2) values, which indicate the proportion of variance in the RMS signal explained by the normalized signal, also exhibit considerable variability. R^2^ values for RMS vs. MEAN, for example, spanned a wide range, from −0.028 for the right bicep to 1.000 for the right tricep, further highlighting the otential for dynamic methods to yield variable results.

In contrast, static normalization methods, such as those based on maximum voluntary contraction (MVC), offer more stable and consistent linear relationships as they rely on an external, fixed reference. However, this stability comes at the cost of reduced task-specificity as static methods may not accurately reflect the changes in muscle activation patterns that are specific to the task being performed. The choice between dynamic and static normalization, therefore, necessitates a careful evaluation of the research question and the acceptable balance between reliability in capturing the overall trend and the desire for consistent amplitude scaling in muscle activation data.

In conclusion, this study highlights the strengths and limitations of different sEMG normalization methods, providing a framework for their application in sports and rehabilitation. No single method universally outperformed the others. CARA normalization is a reliable method for upper-limb muscles, particularly in tasks involving moderate to low effort levels, such as in rehabilitation settings. MEAN normalization excels in dynamic tasks, offering robust preservation of signal characteristics and variance. MVC normalization remains a gold standard for isometric tasks, but its feasibility is limited in clinical or fatigued populations. Future research should build upon these findings by exploring novel normalization techniques, integrating advanced statistical models, and expanding the scope of analysis to include a wider range of muscles and movement patterns. By tailoring normalization strategies to specific contexts, researchers and practitioners can enhance the accuracy and applicability of sEMG analysis, contributing to better outcomes in sports performance, rehabilitation, and clinical assessments.

## Figures and Tables

**Table 2:** Statistical results for different normalization methods across muscles and sides. Values represent mean ± standard deviation for all volunteers. Normalization methods: MVC: maximal voluntary contraction, SMVC: submaximal voluntary contraction, ISOK: isokinetic, CARA: remote voluntary contraction (carpi radialis), DIGI: remote voluntary contraction (digitorum), MEAN: mean signal, and PEAK: peak signal.

Metric	Muscle	Right	Left	Overall	Notes
CCC	Biceps	0.454 ± 0.402	0.587 ± 0.449	0.520 ± 0.426	Best: CARA
	Triceps	−0.023 ± 0.181	0.170 ± 0.366	0.074 ± 0.274	Best: CARA
SPCC	Biceps	0.897 ± 0.026	0.695 ± 0.437	0.796 ± 0.232	Consistent in all
	Triceps	0.913 ± 0.058	0.865 ± 0.036	0.889 ± 0.047	High for CARA/DIGI
$R^{2}$	Biceps	0.793 ± 0.036	0.604 ± 0.401	0.699 ± 0.219	Peak: MEAN
	Triceps	0.846 ± 0.107	0.748 ± 0.085	0.797 ± 0.096	Robust for all
LCC	Biceps	33.07 ± 33.42	39.81 ± 45.10	36.44 ± 39.26	Variability high
	Triceps	91,860 ± 183,600	11.52 ± 10.43	45,935.76 ± 91,805	High in MVC

**Table 3:** Sensitivity and effectiveness of normalization methods vs. previous studies.

Method	Sensitivity (Current Study)	Effectiveness (Current Study)	Comparison to Previous Studies
**CARA**	0.63 ± 0.04	0.82 (left biceps)	Outperformed MVC in dynamic tasks, aligning with Zeller et al. [27]; reduced fatigue bias vs. MVC [16,54].
**SMVC**	−0.02 ± 0.23	0.23 (left biceps)	Matched Burden et al. (1999) findings on submaximal protocols preserving linearity; showed lower sensitivity [6].
**ISOK**	0.28 ± 0.17	0.52 (right biceps)	Consistent with Burden et al. (1999) on isokinetic reliability; less effective in multi-joint tasks [6].
**MVC**	0.30 ± 0.30	0.59 (left biceps)	Aligned with other authors on MVC’s variance explanation; overestimated activation in dynamic tasks [16].
**MEAN**	0.13 ± 0.13	0.37 (left biceps)	Superior variance explanation ($R^{2}=1.0$) vs. MVC; similar to dynamic normalization in [31].
**DIGITORUM**	0.20 ± 0.41	0.69 (right biceps)	Novel approach; no direct precedent. Highlighted stabilizer interference, as in [54].

## Data Availability

The raw data supporting the conclusions of this article will be made available by the authors on request.
